# A Comparative Study of 0.5% and 0.75% Ropivacaine for Supraclavicular Brachial Plexus Block in Upper Limb Surgery

**DOI:** 10.7759/cureus.90727

**Published:** 2025-08-22

**Authors:** Nausheen Ali, Neha Dubey, Shalini Sahu, Anupam Pandey, Mahima Batra

**Affiliations:** 1 Department of Anesthesiology, People's College of Medical Sciences and Research Centre, Bhopal, IND

**Keywords:** anesthesia, monitored anesthesia care, ropivacaine, sensory and motor blockade, supraclavicular brachial plexus block

## Abstract

Background

For surgery of the upper extremity, one method is the supraclavicular block, as it is a reliable anesthetic, safe, and has a rapid onset. Although for epidural anesthesia, ropivacaine has been studied extensively, few reports exist on its use in supraclavicular brachial plexus block in two different concentrations. The present study was done to evaluate the efficacy of 0.5% ropivacaine for supraclavicular brachial plexus block and to compare it with 0.75% ropivacaine for upper limb surgeries in terms of characteristics of supraclavicular blockade.

Methods

A prospective, blinded randomized study was conducted, enrolling 60 patients of either sex, American Society of Anesthesiologists (ASA) I and II, who were allocated into two groups in which supraclavicular brachial plexus block was performed using ropivacaine in concentrations of 0.5% and 0.75%, respectively. The onset and duration of sensory and motor blocks were recorded.

Results

The onset of sensory and motor blockade was faster in patients receiving ropivacaine 0.5% than in those receiving ropivacaine 0.75%. However, the sensory and motor blockade duration was longer in patients who received ropivacaine 0.75%, in comparison to patients who received ropivacaine 0.5%. No statistically significant difference was found in the quality of blocks in both groups.

Conclusion

Ropivacaine 0.75% provides a longer duration of sensory and motor blockade compared to 0.5% when used for supraclavicular brachial plexus block, making it more suitable for prolonged upper limb surgeries and in cases where prolonged pain relief is prioritized. However, 0.5% may be preferable in cases where early motor recovery is desired. Both concentrations were well-tolerated without any significant complications.

## Introduction

Regional anesthesia provides highly effective pain relief by targeting the nerves transmitting pain signals from the surgical site. During peripheral nerve blocks, the anesthesia is localized to the operative site, allowing the patient to maintain control over their airway and breathing and to remain conscious as well [1]. This reduces the probability of complications associated with the administration of general anesthesia. Also, regional anesthesia can lead to reduced opioid consumption postoperatively. This significantly contributes to improving patient comfort and faster recovery, facilitating earlier mobilization and rehabilitation. Aside from the clinical advantages, adopting regional anesthesia in upper limb surgery can lead to significant healthcare cost savings such as reduced hospital stay, faster recovery times, and decreased postoperative complications. The supraclavicular brachial plexus block is favored for ambulatory upper extremity procedures (excluding shoulder surgeries) due to its rapid onset, reliable anesthesia, and safety profile [2].

A successful peripheral nerve block, including a brachial plexus block for upper limb surgery, depends on the choice of local anesthetic (LA) drug. The onset, duration, quality, and possibility of adverse effects of anesthesia are all greatly influenced by the type of LA used during the procedure.

Bupivacaine, a long-acting local anesthetic, is the most commonly used local anesthetic for peripheral nerve blocks because of its long duration of action and high-quality sensory blockade compared to motor blockade [3]. The long-acting local anesthetic ropivacaine is a more recent drug whose neuronal blocking potential in peripheral nerve blockade appears to be on par with or better than that of bupivacaine. According to various studies, it has a far higher safety margin than bupivacaine due to its reduced cardiac and central nervous system toxicity; as a result, it can also be administered at higher dosages. Ropivacaine's weaker motor blockage in comparison to bupivacaine is one of its main disadvantages [4].

One important determinant of the local anesthetic's systemic absorption and toxicity potential is its concentration. Ropivacaine's ability to deliver dependable surgical anesthesia is widely established. However, there is disagreement over the concentration needed to produce this ideal result [5].

A comparative study would provide strong evidence on the most effective concentration for brachial plexus block, ensuring that patients receive the best possible surgical experience. An adequately designed comparative study can provide insights into the balance between pain control and recovery, helping anesthesiologists make informed decisions.

The present study aimed to compare the effects of 0.5% and 0.75% ropivacaine in supraclavicular brachial plexus block for upper limb surgeries. The study was focused on assessing both groups' onset, duration, and characteristics of sensory and motor blockade. The primary outcomes included the onset and duration of sensory and motor blockade, while secondary outcomes encompassed complications, postoperative analgesia consumption, and patient satisfaction.

## Materials and methods

A single-center, hospital-based, prospective randomized comparative study was conducted after obtaining clearance from the Institutional Ethical Committee of People's College of Medical Sciences and Research Centre, Bhopal. Sixty patients of either gender, more than or equal to 18 years of age, with a BMI of more than 25 and undergoing any type of upper limb surgery under supraclavicular plexus block and patients’ physical status of American Society of Anesthesiologists (ASA) Grades I and II were enrolled in this prospective study, while ASA Grade 3 or higher patients with an allergy to ropivacaine, and patients on antiarrhythmic drugs were excluded from this study. The study was carried out for a period of one month, from April 1, 2025, to April 30, 2025.

According to the random sampling method, the patient was allocated to two groups: Group R5 received 0.5% of ropivacaine, and Group R75 received 0.75% of ropivacaine.

Sample size calculation

This study was designed as a preliminary investigation to compare two concentrations of ropivacaine in supraclavicular brachial plexus blocks. The difference between the 50-minute sensory block's total time was used to determine the sample size. Using the formula $n = 2 \left( Z_{\alpha} + Z_{1-\beta} \right)^{2} \times \frac{SD^{2}}{d^{2}}$, the sample size needed to have a 90% chance of detecting a 50-minute decrease in length (level of significance 0.05) was 26 patients per group. We decided to include 30 patients in each group to account for dropout. This figure was thought to be enough for identifying variations in block features that are clinically significant.

After describing the process to the patients in a manner they could understand, informed written consent was acquired. Microsoft Excel's computer-generated random numbers (Microsoft Corporation, Redmond, WA, US) were used for block randomization. Once the patient gave their informed consent, the opaque sealed envelopes containing the random numbers were opened.

All blocks were performed using the classical landmark-guided technique under aseptic precautions as described by Sahu et al. [6]. After painting and draping, a supraclavicular block was given with 150 mg of 0.5% ropivacaine (30 ml) in group R5 patients and 150 mg of 0.75% ropivacaine (20 ml) in group R75 patients using the paresthesia technique. Patients' IV access was secured in both groups with an 18-G cannula on the opposite hand, and IV fluids were started. All patients were premedicated with IV midazolam as a sedative dose of 0.5 mg/kg body weight 20 minutes before giving the block.

Baseline parameters were recorded by attaching all basic monitoring devices. The patients were adequately monitored perioperatively. The duration of surgery was noted. The pinprick method was used to test for the commencement of sensory blocking; the patient was assessed every minute after that until the pinprick caused no pain. The modified Bromage scale was used to evaluate the motor block. Patients were assessed for the length of sensory and motor blockade following surgery, and the time was recorded.

Adapted Modified Bromage scale [3]: Grade 1: Ability to elevate the outstretched arm to 90 degrees for a complete two seconds; Grade 2: Unable to elevate the extended arm but able to move the fingers and flex the elbow; Grade 3: Able to move fingers but unable to flex the elbow; Grade 4: The inability to move the fingers, elbow, or arm.

The onset of motor blockade will be considered when there is a Grade 1 motor blockade. Peak motor block will be regarded as when there is a Grade 3 motor blockade.

All procedures were performed by the same anesthesiologist to maintain uniformity and minimize inter-operator variability.

Statistical analysis

All the data analysis was performed using SPSS version 20 software. Quantitative data were expressed as mean ± standard deviation (SD). Parametric variables were analyzed using the unpaired (independent samples) Student’s t-test. Within-group comparisons, where applicable, were performed using the paired sample t-test. Qualitative (categorical) variables were analyzed using the chi-square test. Non-parametric ordinal data were analyzed using the Mann-Whitney U test. A p-value of <0.05 was considered statistically significant.

## Results

The demographic profiles of patients of both groups, like age, sex, weight, and ASA grade, were not statistically significant, making the two groups similar and comparable, as shown in Table 1.

**Table 1:** Demographic and clinical characteristics of patients in group R5 (0.5% ropivacaine) and group R75 (0.75% ropivacaine) Continuous variables, such as age, weight, and duration of surgery, are expressed as mean ± standard deviation. Categorical variables, such as sex and ASA physical status, are presented as frequencies. Statistical comparisons between the two groups were performed using Student’s t-test for continuous variables and the chi-square test for categorical variables. A p-value of <0.05 was considered statistically significant. ASA: American Society of Anesthesiologists

Parameter	Group R5 (0.5%) (n=30)	Group R75 (0.75%) (n=30)	Test Used	Test Statistic (t / χ²)	P-value
Age (years, Mean ± SD)	35.4 ± 9.2	36.7 ± 8.8	Unpaired t-test	t = 0.61	0.54
Sex (Male/Female)	18 / 12	17 / 13	Chi-square test	χ² = 0.07	0.79
Weight (kg, Mean ± SD)	62.1 ± 5.6	61.4 ± 6.2	Unpaired t-test	t = 0.48	0.63
ASA Grade (I / II)	20 / 10	22 / 8	Chi-square test	χ² = 0.32	0.57

The study outcomes of Groups R5 and R75 are discussed in Table 2.

**Table 2:** Study outcome in the R5 and R75 groups Statistical comparisons between Group R5 (0.5% ropivacaine) and Group R75 (0.75% ropivacaine) were performed using the unpaired (independent) Student’s t-test for normally distributed continuous variables (onset and duration of sensory and motor block, time to rescue analgesia, and total analgesic consumption). A p-value of < 0.05 was considered statistically significant.

		Group R5 (n=30)	Group R75 (n=30)	t-value	P-value
	Onset of Block (in Minutes)				
Sensory Block		16.8 (±2.5)	18.25 (±2.2)	2.89	0.0016
Motor Block		20.7 (±1.5)	21.9 (±1.2)	4.08	< 0.0001
	Duration (in Hours)				
Sensory Block		7.45 (±0.9)	8.50(±1.1)	3.93	< 0.0001
Motor Block		8.50 (1.05)	9.75 (1.20)	4.31	< 0.0001
Rescue Analgesia		10.50 (±1.45)	11.75(±2.05)	3.27	0.0004
Amount of Analgesia (milligrams of Diclofenac)		210 (±40.45)	170 (±25.53)	4.58	< 0.0001

The onset of sensory block was faster in Group R5 (16.8 ± 2.5 minutes) as compared to Group R75 (18.25 ± 2.2 minutes), with a significant p-value. Similarly, the onset of motor block was earlier in Group R5 (20.7 ± 1.5 minutes) than in Group R75 (21.9 ± 1.2 minutes), which was highly significant (p < 0.0001).

The duration of sensory block was longer in Group R75 (8.50 ± 1.1 hours) compared to Group R5 (7.45 ± 0.9 hours), and the duration of motor block was also prolonged in Group R75 (9.75 ± 1.20 hours) versus Group R5 (8.50 ± 1.05 hours), with both differences being statistically significant (p < 0.0001).

The time to first rescue analgesia was significantly longer in Group R75 (11.75 ± 2.05 hours) than in Group R5 (10.50 ± 1.45 hours), with a p-value of 0.0004. Additionally, the median number of analgesic doses required was lower in Group R75 (2 doses) compared to Group R5 (3 doses). The total amount of diclofenac required was also significantly less in Group R75 (170 ± 25.53 mg) than in Group R5 (210 ± 40.45 mg), with a p-value of < 0.0001.

Table 3 demonstrates a non-parametric comparison between R5 and R75.

**Table 3:** Comparison of doses of analgesia between Group R5 and Group R75 This table presents a non-parametric comparison of the number of analgesic doses required in the postoperative period between the two groups. The data are expressed as medians. The Mann–Whitney U test was used due to the ordinal nature of the data. A p-value of < 0.05 was considered statistically significant.

Parameter	Group R5 (n=30)	Group R75 (n=30)	Test Used	Test Statistic	P-value
Doses of Analgesia (Median)	3	2	Mann–Whitney U test	U = 287.0	0.0021

These findings suggest that Group R75 experienced a longer duration of analgesia with reduced analgesic requirements, although the block onset was slightly delayed compared to Group R5.

Hemodynamic parameters

Heart rate, systolic and diastolic blood pressure, mean arterial pressure, and peripheral oxygen saturation in percentage (SpO2) were all normal in both groups during the procedure and after the block; no intervention was necessary, and the differences between the two groups were not statistically significant.

## Discussion

A brachial plexus block has long been regarded as a safe procedure when done correctly, which includes patient selection and monitoring. Brachial plexus blockade, however, may create a possible site for local anesthetic absorption and the emergence of systemic toxicity, as it is a vascular area. Long-acting bupivacaine is the most commonly used local anesthetic for supraclavicular block in patients undergoing elective upper limb procedures. However, the CNS and cardiovascular system (CVS) side effects are among its drawbacks. The result of a thorough search for a less harmful substitute for bupivacaine is ropivacaine [7].

Despite being safe, ropivacaine has been shown to be less effective than bupivacaine, act for a slightly shorter duration, and have some motor-sparing properties [8]. Much research has been done on ropivacaine as a labor analgesic, and it has been shown to be just as effective as bupivacaine with minimal side effects [9,10].

To determine the ideal concentration for the supraclavicular brachial plexus block, we have attempted this study with ropivacaine.

In the current study, the total dose of ropivacaine was equal in both groups, i.e., 150 mg. Only the concentration (0.5% versus 0.75%) and volume of the drug administered differed in the two groups. The onset of sensory block occurred significantly faster in Group R5 compared to Group R75. Similarly, the onset of motor block was also earlier in Group R5. This implies that the high volume of LA, rather than concentration, determines the onset of motor and sensory block in the current study. This could have implications for the speed of anesthesia onset and its potential effect on patients' comfort and readiness for procedures. Group R75 exhibited longer durations for sensory and motor blocks than Group R5. This suggests that while the higher concentration takes longer to induce the block, it maintains it for an extended period.

The studies done by Klein et al. and Bertini et al. found that raising the ropivacaine concentration from 0.5% to 0.75% did not improve the interscalene brachial plexus block's onset or duration [11,12]. However, Casati et al. found that ropivacaine, at a dose of 2.5-2.6 mg/kg, was a safe and effective local anesthetic for brachial plexus block with no negative side effects [13].

In the current study, the difference in onset of sensory and motor blockade in both groups was found to be statistically significant (p˂0.05). Similarly, in studies done by Ana A et al. and Modak S et al., similar results were observed [14,15].

Kaur et al. compared ropivacaine with bupivacaine for axillary brachial plexus block and observed that ropivacaine showed a comparatively better quality of analgesia in comparison to bupivacaine and with a shorter onset (5 min for 0.5% ropivacaine compared to 20 min for bupivacaine). Also, recovery time for both sensory and motor blockade was shorter with ropivacaine in comparison to bupivacaine [16].

The longer duration in our study could be beneficial in surgeries or procedures requiring prolonged anesthesia effects, potentially reducing the need for additional interventions or top-up doses. Tailoring doses based on the procedure type and patient needs could enhance patient comfort.

In the current study as well as in the literature, the clinical results of ropivacaine in brachial plexus blocks indicate that the onset, duration, and quality of blockade are almost similar to bupivacaine. However, a study done by Venkatesh RR et al. found that increasing the concentration of ropivacaine from 0.5% to 0.75% failed to improve onset and duration [5]. This difference is maybe due to different methodologies, block approaches, or sample sizes.

Cacciapuoti A et al. reported that both sensory and motor onset times were faster with 0.75% ropivacaine (7.5±1.2 min and 14.0±2.3 min, respectively) [17].

The findings in the current study suggest that the higher dose leads to a slower onset but a longer duration of block, potentially reducing the need for repeated interventions during longer procedures and improving overall patient comfort. The reduced need for rescue analgesia and lower overall analgesic consumption associated with the higher concentration (R75) imply better postoperative pain control. Better patient outcomes, a speedier recovery, and possibly fewer adverse effects linked to the overuse of analgesics could result from this. Optimizing resource utilization in healthcare settings may be possible with an understanding of how various doses affect analgesic requirements. Using doses that lead to lower analgesic consumption may have cost-saving implications and reduce the workload on healthcare providers.

Limitations of the study

This study was conducted using a landmark-guided technique, which may have inherent variability compared to ultrasound guidance. Although all procedures were performed by a single anesthesiologist to maintain consistency, the absence of imaging may limit reproducibility in less experienced hands. The study was performed at a single center with a relatively small sample size, which may limit generalizability. Future studies using ultrasound guidance and multicenter designs are warranted to strengthen the findings. Also, the duration of postoperative follow-up was limited to the early postoperative period; hence, long-term effects or complications could not be evaluated.

## Conclusions

Overall, these findings highlight that lower concentration induces blocks more rapidly but with shorter durations, while the higher concentration takes longer to induce blocks but sustains them for an extended period. Additionally, the higher dose seems to have advantages in terms of reducing rescue analgesia requirements and overall analgesic consumption.

An anesthesiologist might opt for the lower dose (R5) when rapid onset of anesthesia is required, such as in emergencies or procedures where quick patient turnover is crucial. Conversely, the higher concentration (R75) could be preferred for longer procedures where sustained anesthesia and prolonged pain relief are prioritized.
